# Large dependency of intracellular NAD and CoA pools on cultivation conditions in *Saccharomyces cerevisiae*

**DOI:** 10.1186/s13104-021-05783-6

**Published:** 2021-09-23

**Authors:** Kanhaiya Kumar, Per Bruheim

**Affiliations:** Department of Biotechnology and Food Science, Norwegian University of Science and Technology, Sem Sælands vei 6/8, N-7491 Trondheim, Norway

**Keywords:** *Saccharomyces cerevisiae*, Metabolomics, Mass spectrometry, Bioreactor, NAD, CoA, 13C internal standard

## Abstract

**Objective:**

The objective of this study was to investigate the variation of NAD and CoA metabolite pools in *Saccharomyces cerevisiae* cultivated under various cultivation conditions. This study complements a previous report on glycolytic, pentose phosphate pathway, tricarboxylic acid cycle, amino acids, and deoxy-/nucleoside phosphate pools determined under the same cultivation conditions.

**Results:**

*S. cerevisiae* pellets from batch (four carbohydrate sources) and chemostat (carbon-, nitrogen-, phosphate—limited and a range of dilution rates) bioreactor cultivations were extracted and analyzed with two recently established absolute quantitative liquid chromatography mass spectrometry (LC–MS/MS) methods for NAD and CoA metabolites. Both methods apply ^13^C internal standard dilution strategy for the enhanced analytical accuracy and precision. Individual metabolite pools were relatively constant for the different growth rates within the same mode of cultivation, but large differences were observed among some of the modes, i.e. NAD metabolites were 10 to 100-fold lower in nitrogen limited chemostats compared to the other modes, and phosphate limited chemostats were characterized with much lower CoA metabolite pools. The results complement the previous results and together provide a comprehensive insight into primary metabolite pools variations at a large range in growth and carbon source consumption rates.

**Supplementary Information:**

The online version contains supplementary material available at 10.1186/s13104-021-05783-6.

## Introduction

The baker’s yeast *Saccharomyces cerevisiae* is one of the most studied biological model systems, from basic mechanistic studies to applied focus and usage. While there are many high-quality data sets on transcriptome and proteome levels, the few reports on quantitative metabolite profiling present a large variability in concentrations, and also an incomplete coverage of central metabolism [1–8]. The reasons are multiple; different strains and cultivation conditions, different sampling and analytical protocols all contribute to this inter- laboratory variability. Central research groups in the global metabolomics community have initiated an important focus on standardization and model organisms [9, 10]. Our group has over some years developed mass spectrometry (MS) based quantitative metabolite profiling methods applying ^13^C internal standard methods and applied it on several model systems [11–17]. These methodologies have until recently covered glycolytic (EMP), pentose phosphate pathway (PPP), tricarboxylic acid cycle (TCA) and other organic acids, amino acids, and complete deoxy-/nucleoside phosphate pools. Evaluation and interpretation of metabolite pool data are challenging, the experimental design is often multi-variable where differences in growth and substrate consumption vary even though the study aims to explore e.g. strain differences or response to stressors. This was the background for undertaking a large study on metabolite pool variability in twenty-two different cultivation conditions [18]. Later we have established absolute quantitative MS-based profiling of NAD and CoA metabolite classes using ^13^C internal standard strategy [19, 20]. Here we revisit the previous cultivations and analysed samples with the two latter methods that when merged with the previous data together provide quite complete and comprehensive coverage of central carbon-, energy- and redox metabolism of *S. cerevisiae* under a range of cultivation conditions.

## Main text

### Methods

#### Cultivation and analysis

Detailed protocols for medium and cultivation conditions can be found in the previous publication presenting intracellular pools of EMP, PPP, TCA, amino and organic acids, and complete deoxy-/nucleoside phosphate pools [18]. *Saccharomyces cerevisiae* CEN.PK 113-7D was grown in the controlled bioreactor in Verduyn medium at pH 5, 30 °C, and maintaining a minimum dissolved oxygen level of 40%. The batch study was conducted in a bioreactor (1500 mL working volume) with four different carbon sources (glucose, fructose, sucrose, and galactose) each was 10 g L^−1^. The strain was also cultivated in a chemostat in a bioreactor (1000 mL working volume) having the same experimental conditions as in a batch but in nutrient (carbon, nitrogen, phosphate) limited conditions. The feed tank of nitrogen and phosphate limited chemostat were containing 0.2 g L^−1^ (NH_4_)_2_SO_4_, 0.02 g L^−1^ KH_2_PO_4_, respectively, whereas the carbon limited chemostat was run in low glucose (LG, 1.0 g L^−1^), and high glucose (HG, 10.0 g L^−1^) medium. The two LC–MS methods used here were established after the former study and are presented in detail in the two recent publications, for CoA metabolites [19] and NAD metabolites [20], respectively. Of particular importance for these two metabolite groups is the high instability, especially of reduced compounds. Thus, the standard sampling protocols with fast filtration, freeze–thaw extraction, and concentration by freeze-drying can’t be applied, but pelleting using centrifugation and snap freezing had to be used. Pellets for NAD analysis were extracted in acetonitrile:methanol:water (60:20:20 v/v) with 15 mM ammonium acetate pH 9.7, while pellets for CoA analysis were extracted in acetonitrile:methanol:water (70:10:20, v/v/v) containing 50 mM ammonium acetate pH 5.0 (which are the mobile phase compositions at the start of the injection). It is strictly necessary to extract samples and run LC–MS analysis within the next 10–15 h. ^13^C-labeled *S. cerevisiae* extracts are spiked into the extraction solvent before the extraction of cell pellets. This is necessary since both methods are analytically challenging with many parameters compromising accuracy and precision [19], and the ionization efficiencies between oxidized (NAD, NADP) and reduced (NADH, NADPH) is over 50 times different.

### Results and discussion

**Fig. 1:**
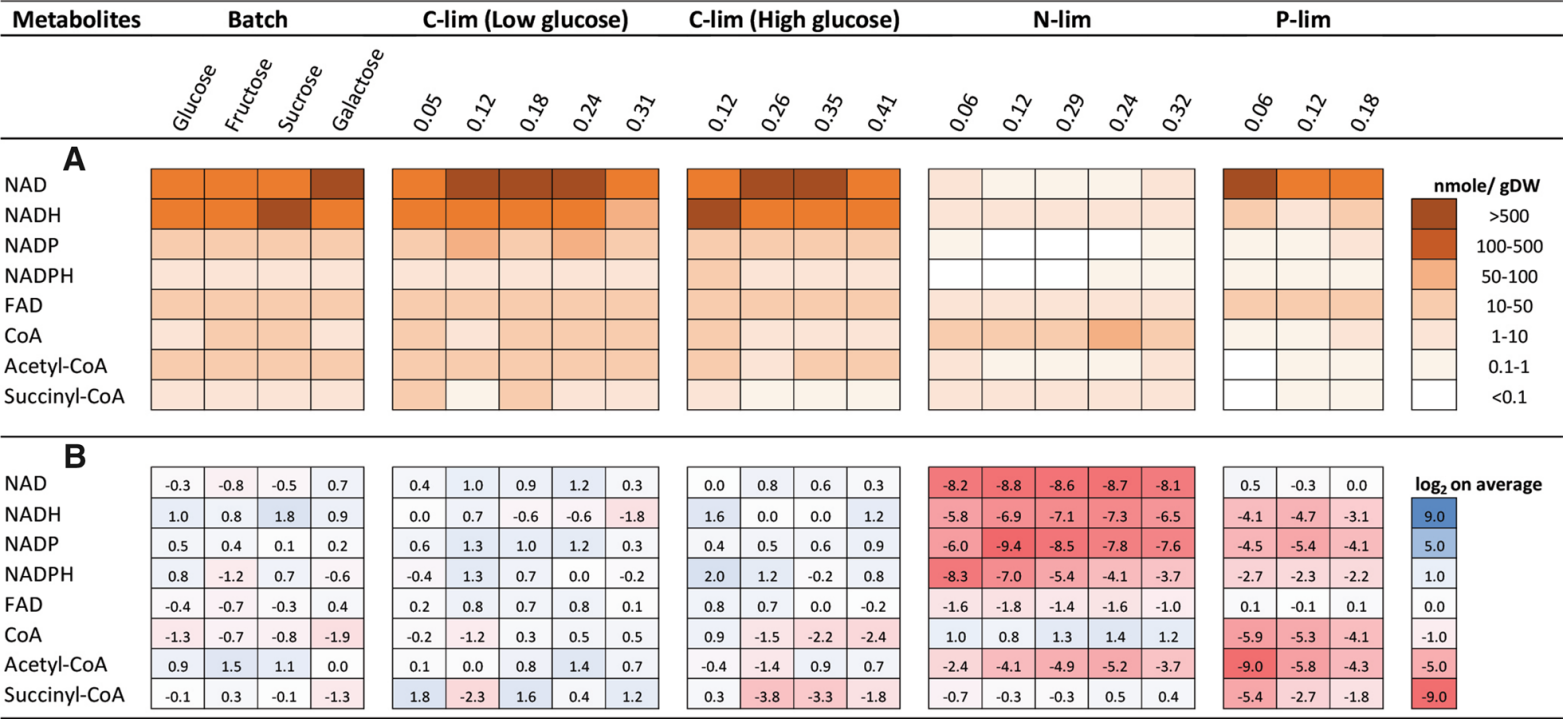
Intracellular concentrations (in nmoles/ gDW) of the eight metabolites in the different *S. cerevisiae* cultivations: batch with four different carbon sources, two different glucose-limited chemostats (high and low glucose concentration), nitrogen (N-lim), and phosphate limited (P-lim) chemostats, all chemostats with a range of dilution rates in h^−1^ indicated in the heading (**A**). The same data set but presented as log2 ratios between individual conditions to average for respective metabolite (**B**). Results from two HILIC-MS/MS methods are merged in this heat map presentation. Presented values are the average of two replicas (Additional file 1: Table S1)

Twenty-one different cultivations were run and sampled for the NADs and CoAs analyses. Both HILIC-MS/MS methods return absolute concentrations (here nmole/gDW); thus, the results can be merged and presented together (Fig. 1). The NAD/NADH pair is present at much higher intracellular concentrations than the other metabolites for four of the five modes of operation. All nitrogen-limited (N-lim) chemostats have much lower NAD and NADH concentrations even though they were run at the same range of growth rates, i.e. dilution rates. The phosphate (P-lim) chemostats stand out with the lower NADH to NAD ratio compared to batch and glucose-limited chemostats. Contrary to the NAD metabolites, the CoA metabolites are at the same levels for the N-lim chemostats as for the batch and glucose-limited chemostats. But much lower levels of CoA metabolites are observed for the P-lim chemostats. In all, quite interesting patterns of how NAD and CoA metabolite pools vary with the mode of operation are observed. This preliminary investigation reveals no correlation between growth rate and pool sizes; contrary, the overall picture is that these pools are heavily dependent on cultivation modes and nutrient limitations. There are no entries for the CoA metabolites in the Yeast Metabolome Database (ymdb.ca), but some for the NAD metabolites [6]. The latter vary with large standard deviations but showing the same trends as presented here with 2–10 times higher concentrations of NAD/ NADH compared to NADP/NADPH.

A further inspection at the individual metabolite levels by heat map visualization of the ratio between individual conditions to the average (log_2_ transformed) confirms the impression given in the absolute concentration plot. Also, the NADP and NADPH pools are strongly down-regulated in N-lim conditions (Fig. 1B). The NAD/NADH ratio is much higher in P-lim conditions and the NADP/ NADPH pools are similarly down-regulated as in the N-limited chemostats. The N-lim conditions is able to support CoA levels at the same levels as batch and C-lim chemostats, but the Acetyl-CoA pool is strongly down-regulated. All CoA pools are strongly downregulated in the P-limited state, while FAD seems to be metabolite with the least variations among the cultivation conditions.

In the previous study, a few metabolites were observed to correlate strongly with the growth rate, not necessarily on the global data set but sub-sets on individual modes of operation [18], but most metabolite pools did not exhibit any form of correlation with the growth rate. We can directly out of Fig. 1 conclude on no correlation between metabolite pools and growth rate for the global data set and not for individual cultivation conditions either. Similar tests were also performed against the carbon source consumption rates, which could potentially be linked more directly to pool sizes and intracellular metabolic fluxes [21]. *S. cerevisiae* is a Crabtree positive yeast and ethanol production was observed in most, but not all, of the cultivation conditions [18]. This will also complicate the evaluation and interpretation of the data. Any correlation with carbon source consumption rate was also tested for the current data. Fructose 1,6-bisphosphate data from the previous study were included in this correlation analysis since this metabolite exhibited a positive correlation for certain cultivation modes and it is considered to be a global regulatory metabolite [22]. Slopes and r square values were also calculated and evaluated but did not reveal any trend in the correlation between NAD and CoA metabolites and carbon source consumption, neither in the global nor sub-data sets (Additional file 2: Table S2). A weak correlation was observed between a decrease in NAD concentration and ethanol production (at higher growth rates in the high glucose chemostat and all growth rates in the N-lim chemostat).

Interestingly, the recognized glycolytic regulatory metabolite F-1,6BP does not positively correlate with carbon source consumption for all modes of operation [18], but the interplay of pool sizes and intracellular metabolic fluxes is complex and far from understood. Regulation of fluxes through central pathways (EMP, PPP, TCA) is differently designed than linear biosynthetic pathways. One main reason is that the former serves both for the generation of precursor metabolites and energy (ATP, NADPH) while the latter mostly can shut down via feedback inhibition when the endpoint metabolite accumulates. Based on the recognition that various metabolites serve various roles, various trends in pool size fluctuations are to be expected. ATP and NAD(P)(H) interconnect metabolic networks since they are formed and consumed at multiple sites. Thus, it is interesting to note that these NAD and CoA pools are rather constant for a large range of growth/substrate uptake rates for the same mode of operation, although there are large pool size differences among the modes and that the yeast is able to maintain same growth rate and carbon source consumption even if NAD and CoA metabolites vary with several orders of magnitude. The biomass composition changes with the growth rate, in particular, increased RNA content with increased growth rate [23]. Consumption of monomers increases also with the growth rate. Protein is the main macromolecule (up to 50%) and amino acid biosynthesis consumes NADPH while the polymerization peptide chain is a high consumer of ATP. Still, it is observed quite constant pools of NADPH and ATP (and Energy charge [18]) from low to high growth/substrate consumption rates, implying that the yeast has homeostatic mechanisms for these energy careers and that other metabolite types are permitted to fluctuate, some with potential roles of both measuring and regulating the metabolic flux [22, 24]. Similar to the previous report, NAD pools were found to be dependent upon the medium composition and adjust themselves according to cellular metabolic demand [25, 26]. Tryptophan and aspartate are the precursor for the pyridine nucleotide synthesis. The low NAD metabolite pools in this study are in resonance with the lower level of tryptophan in N- and P-lim as reported in our previous report [18]. Aspartate does not seem to be limiting for the pyridine nucleotide synthesis.

### Conclusions

The latest versions of quantitative LC–MS/MS methodology have been applied for and show a strong cultivation condition dependency of NAD and CoA metabolite pools in *S. cerevisiae*. Especially nitrogen and phosphate limited conditions resulted in down regulation of NAD and CoA metabolite pools.

## Limitations

The quantification of NADs and CoAs is inherently challenging using LC–MS/MS. Besides these, the small sample size (two replicas) is the main limitation of the study since it was based on leftover samples from a large study with 22 different cultivation conditions. But, the variations are small and the measured levels are considered to be valid as there are solid trends in the data sets.

## Supplementary Information

**Additional file 1:** Intracellular concentration of the NAD and CoA metabolites determined with two LC-MS/MS methods.

**Additional file 2:** Correlation analysis of intracellular metabolite concentrations and cultivation data.

## Data Availability

The datasets used and analysed during the current study are available from the corresponding author on reasonable request. The calculated concentrations (and normalized to nmol/g DW) of individual replicas presented in Additional file 1: Table S1 can be merged with the data from the previous study [18].

## Abbreviations

LC–MS/MS: Liquid chromatography–tandem mass spectrometry
MS: Mass spectrometry
EMP: Glycolytic pathway
PPP: Pentose phosphate pathway
TCA: Tricarboxylic acid cycle
*S.**cerevisiae*: *Saccharomyces* *cerevisiae*
P-lim: Phosphate limited chemostats
N-lim: Nitrogen limited chemostats
